# Identification of a novel spliced variant of the SYT gene expressed in normal tissues and in synovial sarcoma

**DOI:** 10.1054/bjoc.2000.1710

**Published:** 2001-04

**Authors:** E Tamborini, V Agus, A Mezzelani, C Riva, G Sozzi, A Azzarelli, M A Pierotti, S Pilotti

**Affiliations:** Department of Pathology, Istituto Nazionale per lo Studio e la Cura dei Tumori, Via G. Venezian, Milano, 1 20133, Italy; Department of Experimental Oncology, Istituto Nazionale per lo Studio e la Cura dei Tumori, Via G. Venezian, Milano, 1 20133, Italy; Department of Musculo-Skeletal Surgery, Istituto Nazionale per lo Studio e la Cura dei Tumori, Via G. Venezian, Milano, 1 20133, Italy

**Keywords:** synovial sarcoma, fusion transcript, RT-PCR analysis, alternative splicing event

## Abstract

Synovial sarcoma (SS) is cytogenetically characterized by the translocation t(X;18)(p11.2-q11.2) generating a fusion between the SYT gene on chromosome 18 and one member of the SSX family gene (SSX1; SSX2; SSX4) on chromosome X. Here, we report for the first time that 2 forms of SYT mRNA are present in both normal tissues and SSs. By amplifying the full-length SYT cDNA of two SSs, we detected 2 bands, here designated N-SYT and I-SYT. The latter, previously undescribed, contains an in-frame insertion of 93 bp. Its sequencing revealed a 100% homology with the mouse SYT gene. These two SYT forms were present, although in different amounts, in all human normal tissues examined, including kidney, stomach, lung, colon, liver and synovia. Coexistence of N-SYT and I-SYT (both fused with SSX) was detected in a series of 59 SSs (35 monophasic and 24 biphasic) and in a SS cell line. A preliminary analysis of the differential expression levels of N-SYT and I-SYT in SSs revealed that the latter was consistently overexpressed, suggesting a role in SS pathogenesis. © 2001 Cancer Research Campaign http://www.bjcancer.com


British Journal of Cancer (2001) 84(8), 1087–1094
© 2001 Cancer Research Campaign
doi: 10.1054/ bjoc.2001.1710, available online at http://www.idealibrary.com on IDEAL®

### Identification of a novel spliced variant of the SYT gene expressed in normal tissues and in synovial sarcoma

**E Tamborini[1], V Agus[1], A Mezzelani[1], C Riva[1], G Sozzi[2], A Azzarelli[3], MA Pierotti[2] and S Pilotti[1]**

[1]Department of Pathology, [2]Department of Experimental Oncology, [3]Department of Musculo-Skeletal Surgery, Istituto Nazionale per lo Studio e la Cura dei Tumori, Via G. Venezian, 1 20133 Milano, Italy

**Summary** Synovial sarcoma (SS) is cytogenetically characterized by the translocation t(X;18)(p11.2-q11.2) generating a fusion between the SYT gene on chromosome 18 and one member of the SSX family gene (SSX1; SSX2; SSX4) on chromosome X. Here, we report for the first time that 2 forms of SYT mRNA are present in both normal tissues and SSs. By amplifying the full-length SYT cDNA of two SSs, we detected 2 bands, here designated N-SYT and I-SYT. The latter, previously undescribed, contains an in-frame insertion of 93 bp. Its sequencing revealed a 100% homology with the mouse SYT gene. These two SYT forms were present, although in different amounts, in all human normal tissues examined, including kidney, stomach, lung, colon, liver and synovia. Coexistence of N-SYT and I-SYT (both fused with SSX) was detected in a series of 59 SSs (35 monophasic and 24 biphasic) and in a SS cell line. A preliminary analysis of the differential expression levels of N-SYT and I-SYT in SSs revealed that the latter was consistently overexpressed, suggesting a role in SS pathogenesis. © 2001 Cancer Research Campaign http://www.bjcancer.com

**Keywords**: synovial sarcoma; fusion transcript; RT-PCR analysis; alternative splicing event

Synovial sarcoma (SS), which accounts for approximately 10% of soft tissue sarcomas, mostly affects young adults (Enzinger and Weiss 1995). It develops mainly in the juxta-articular regions in close association with tendon sheaths, and occasionally in the abdominal wall and head and neck regions (Fetsh and Meis 1993; Miloro et al, 1994; Zeren et al, 1995), lung and pleura (Hisaoka et al, 1998; Nicholson et al, 1998) and several other sites (Cielette et al, 1994; Nielsen et al, 1996; Fritsch et al, 2000). Histologically, a biphasic variant, composed of varying proportions of epithelial and spindle cells and a monophasic variant, containing exclusively spindle cells, are recognized (Fisher, 1986).

Cytogenetically, SS is characterized by the non-random translocation t(X;18) (p11.2-q11.2) (Turc-Carel et al, 1987), which generates the fusion of 2 genes, SYT on chromosome 18 and SSX on chromosome Xp11.2 (Clark et al, 1994), where 5 highly homologous genes (SSX1 to SSX5) have been so far described (Crew et al, 1995; De Leeuw et al, 1996). At present, 3 different types of translocations have been identified in SS, involving SSX1, SSX2 (Crew et al, 1995; de Leeuw et al, 1995) and SSX4 (Skytting et al, 1999). A strong association between SYT-SSX fusion type and morphology has been documented (Kawai et al, 1998; Nilsson et al, 1999; Antonescu et al, 2000), even if exceptions occur (Crew et al, 1995; Kashima et al, 1997; Tsuji et al, 1998; Mancuso et al, 2000).

The SYT gene is expressed in a variety of embryonic and adult tissues (de Bruijn et al, 1996). The corresponding protein, composed by 387 amino acids, is rich in glutamine (19%), proline (16%) and glycine (14%). Comparison database studies have shown the presence of 3 putative SH2 and one SH3 binding domains and an annexin-like imperfect direct repeat (Clark et al, 1994; de Bruijn et al, 1996). Even if no recognizable DNA-binding domain is detectable, it has been recently demonstrated that SYT protein is able to transactivate transcription when targeted to a reporter gene (Brett et al, 1997). This transcriptional role is supported by immunofluorescence experiments using polyclonal antibodies raised against the SYT protein alone or fused with one of the SSX genes, showing that these proteins display a nuclear localization (Brett et al, 1997; Dos Santos et al, 1997).

The expression pattern of the SSX gene group is more restricted: SSX1 and SSX2 transcripts are abundant in human adult testis, expressed at low levels in the thyroid, and not detectable in other normal tissues (Crew et al, 1995; Gure et al, 1997). The encoded proteins of 188 amino acids share 76% identity and 83% similarity and display, like SYT protein, a nuclear localization. Some tyrosine phosphorylation sites have also been detected (Crew et al, 1995; De Leeuw et al, 1996).

SSX proteins have no defined DNA-binding sequence, but contain 2 well-preserved areas displaying a repressor activity, one resembling Krüppel-associated box and the other (named SSXRD) located in the COOH-terminal region. SSX proteins, in fact, can act as transcriptional repressors when fused to a reporter gene (Brett et al, 1997; Lim et al, 1998). However, the Krüppel-associated box-related domain is not retained following the fusion with SYT gene: in the chimaeric proteins the 8 C terminal amino acids of SYT are replaced by 78 residues of SSX genes. The aberrant SYT-SSX proteins could affect cell function and lead to neoplasia perhaps as a result of transactivation of other target genes not normally transcribed.

Here we demonstrate that SYT mRNA is present in 2 forms one of which, heretofore undescribed, contains an in-frame insertion of 93 bp sharing a 100% homology with the corresponding mouse SYT gene. Coexistence of N-SYT and I-SYT, both fused with SSX, was detected in a series of 59 SSs and in a SS cell line. Our data suggest a critical role of the new I-SYT form in SS pathogenesis.

*Received 4 September 2000*
*Revised 9 January 2001*
*Accepted 11 January 2001*

*Correspondence to:* E Tamborini

### MATERIALS AND METHODS

### Tumours and patients

59 SSs were analysed by RT-PCR on frozen material. 31 showed the non-random translocation SYT-SSX1, 16 the SYT-SSX2 and one the SYT-SSX4; in the remaining 11 tumours no specific fusion transcript has been yet detected and these are the subject of further studies. Among the SYT-SSX1 tumours, 16/31 (51.6%) were monophasic and 15/31 (48.4%) biphasic; in the SYT-SSX2 group 12/16 (75%) were monophasic and 4/16 (25%) biphasic. The single SYT-SSX4 tumour was monophasic. There were 21 primary tumours, 15 local relapses and 23 metastases; in 2 cases (patients n. 27 and 42) we analysed both the primary tumour and the metastasis, and in 4 cases (patients n. 25, 28, 41 and 55) we analysed relapses and/or metastases (Table 2).

The human synovial sarcoma CME cell line was kindly provided by Dr B Kazmierczak (Renwick et al, 1995); these cells had the typical (X;18) translocation and showed the SYT-SSX2 fusion transcript.

6 normal tissue samples (kidney, stomach, liver, lung, colon and synovia) derived from different patients surgically treated in our Institute were also analysed.

### RNA extraction and reverse-transcription reaction (RT-PCR)

Total RNA was extracted using the RNAzol method (GIBCO BRL, Life Technology) from snap-frozen tissue samples stored at –80˚C. One µg of RNA was reverse-transcribed to cDNA with oligo(dT) and random examers primers, using Superscript II reverse transcriptase (GIBCO BRL, Paisley, UK), according to the manufacturer's conditions. One µl of cDNA was used as template for each PCR reaction, which was performed using AmpliTaq (Perkin Elmer), according to the manufacturer's conditions. All the amplification products were stained with ethidium bromide and analysed on 1.8% agarose gel.

### SYT-SSX PCR

The detection of the putative SYT-SSX1 or SYT-SSX2 fusion transcript was carried out with the following primers:

SYT: 5′-CAACAGCAAGATGCATACCA-3′
SSX1: 5′-GGTGCAGTTGTTTCCCATCG-3′
SSX2: 5′-GGCACAGCTCTTTCCCATCA-3′

PCR conditions were: 94˚C for 3 minutes, 35 cycles of denaturation at 94˚C for 30 s, annealing at 58˚C for 30 s and elongation at 72˚C for 30 s; 5 minutes of terminal elongation at 72˚C (Kawai et al, 1998).

The detection of the fusion transcript SYT-SSX4 was performed by a nested PCR using the following primer pairs:

SYT external: 5′-CAACAGCAAGATGCATACCA-3′
SSX external: 5′-TGCTATGCACCTGATGACGA-3′

The annealing temperature was 52˚C for 1 min.

SYT internal: 5′-AGACCAACACAGCCTGGACCA-3′
SSX4 internal: 5′-GGCACAGCTGTTTCCCATCA-3′

The annealing temperature was 58˚C for 1 min (Skytting et al, 1999). Patients n. 30 (A) and n. 13 (B) were chosen to amplify the full-length cDNA of both SYT-SSX1 (patient A) and SYT-SSX2 (patient B), using GOLD AmpliTaq and the following primers:

SYT FL F: 5′-TGG ATG GGC AAC ATG TC-3′
SSX1 FL R: 5′-CTC ATC AAG GGC ATG TGT CGT AT-3′
SSX2 FL R: 5′-TGG GCA TGT GTC GTA TCC CTG AGG-3′

PCR conditions were: 96˚C for 8 min, 35 cycles of denaturation at 94˚C for 30 s, annealing at 50˚C for 30 s and elongation at 72˚C for 2 m; 5 min of terminal elongation at 72˚C.

### Cloning into pGEM-T vector

PCR products of full-length SYT-SSX1 and SYT-SSX2 were cloned into pGEM-T vector (Promega), according to the manufacturer's conditions. After ligation and transformation into competent *E. coli* DH5 alfa cells, recombinant clones were isolated by PCR screening (using T7 and SP6 universal primers) and subsequently entirely sequenced.

### DNA sequencing

All the sequence reactions were carried out using an automated sequencing system (377 DNA Sequencer, ABI PRISM PE, Applied Biosystem) following standard protocols.

### Internal PCR

We used an upstream 5′ primer to amplify the fusion transcript (SYT-SSX1 or SYT-SSX2) including a bigger fragment of the 5′ portion of SYT gene:

SYT 500 F: 5′-GCC ATC ATC ACA GAG CAT GC-3′
SSX1: 5′-GGT GCA GTT TCC CAT CG-3′
SSX2: 5′-GGC ACA GCT CTT TCC CAT CA-3′

PCR conditions were: 94˚C for 3 min, 25–35 cycles of denaturation at 94˚C for 30 s, annealing at 56˚C for 30 s and elongation at 72˚C for 1 min; 5 min of terminal elongation at 72˚C.

Normal tissues SYT cDNA was amplified using GOLD AmpliTaq and the following primers:

SYT 500 F: 5′-GCC ATC ATC ACA GAG CAT GC-3′
SYT 1000 R: 5′-CTG TCC TGG GTA ACC TTG CTG CCC-3′

PCR conditions were: 96˚C for 8 min, 25 to 35 cycles of denaturation at 94˚C for 30 s, annealing at 56˚C for 30 s and elongation at 72˚C for 1 min; 5 min of terminal elongation at 72˚C.

SYT 500 F and SYT 1000 R primers corresponds to bp 515–535 and 981–1005 of SYT cDNA sequence (GenBank Accession n. X79201).

### Densitometric analysis

PCR reactions used for densitometric analysis were performed at different total cycles (25, 30 and 35). Samples were run in a 1.8% agarose gel and analysed by Image Master VDS Scan Program

© 2001 Cancer Research Campaign

**Table 1** Densitometrical analysis on normal tissues

| | Kidney | Stomach | Liver | Lung | Colon | Synovia |
|---|---|---|---|---|---|---|
| %I-SYT | 25.9 | 11.2 | 14.5 | 14.1 | 11.8 | 15.9 |
| %N-SYT | 74.1 | 88.8 | 85.5 | 85.9 | 88.2 | 84.1 |
| Total | 100.0 | 100.0 | 100.0 | 100.0 | 100.0 | 100.0 |
| I-SYT/N-SYT | 0.35 | 0.12 | 0.17 | 0.16 | 0.13 | 0.19 |

(Pharmacia, Amersham). For densitometric analysis samples where a PCR saturation was detected, were avoided. Each lane was analysed separately and the numeric value obtained by the scanning of the electrophoretically higher band was divided by the value of the lower one. The calculated ratio between the software value of I-SYT and N-SYT for each PCR reaction is reported in column R of Table 2 and was performed on only 39/59 tumours and CME synovial sarcoma cell line cDNAs.

### RESULTS

### Detection of two forms of SYT gene

By amplifying the full-length cDNA of transcripts SYT-SSX1 (Table 1, case n. 13) and SYT-SSX2 (Table 1, case no. 30) two bands for each amplification reaction were detected (Figure 1A). To avoid cross reactivity during the sequencing procedures and to verify their specificity, we cloned the 2 bands obtained from each patient into pGEM-T vector. After PCR screening, we selected clones containing 2 different inserts for each type of fusion transcript. The subsequent sequencing revealed that the 2 lower bands of 1390 bp (one from SYT-SSX1 and one from SYT-SSX2) had a 100% homology with the already described fusion transcripts, while the higher ones showed a novel sequence, corresponding to an in-frame insertion of 93 bp in position n. 896 of the SYT gene (GenBank Accession AF244972). Comparative analysis of this 93 bp inserted sequence, responsible for the addition of 31 residues, revealed a 100% homology with the mouse SYT gene (Figure 1B). After amplification with the same primer pairs for the mouse SYT gene, from mouse liver and lung cDNA (kindly provided by Dr Manenti), we detected the same two bands observed in humans, as confirmed by sequencing procedures (Figure 1C).

### Analysis of a series of human normal tissues

In order to verify if these 2 SYT forms were also present in normal tissues cDNAs, we performed a PCR amplifying the SYT gene only, using SYT500F and SYT1000R as primer pairs (Figure 2A). A control PCR was done on an housekeeping gene, beta actin (data not shown). In all the analysed tissues (Figure 2B), 2 bands of 583 and 490 bp were detected. Their sequencing revealed that the former correspond to the new SYT form (here named I-SYT), while the latter corresponded to the already described SYT gene (here named N-SYT).

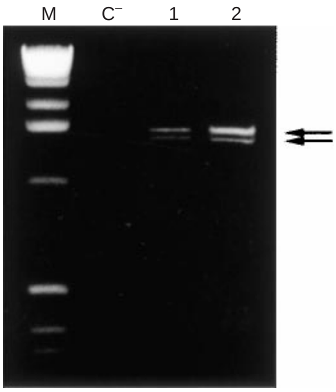

**Figure 1** (**A**) RT-PCR of the full length cDNA of two synovial sarcomas. M = molecular weight marker. 1 = Case no. 13 of Table 2 (SYT-SSX1). 2 = Case no. 30 of Table 2 (SYT-SSX2). C⁻= no DNA was added to the PCR reaction.
(**B**) Aminoacidic translation of I-SYT and N-SYT.

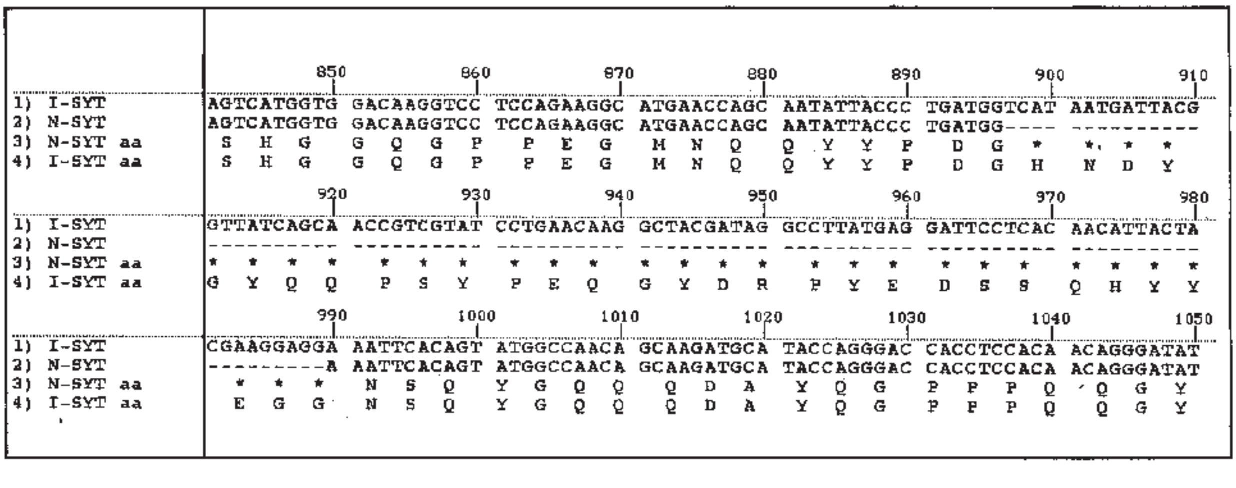

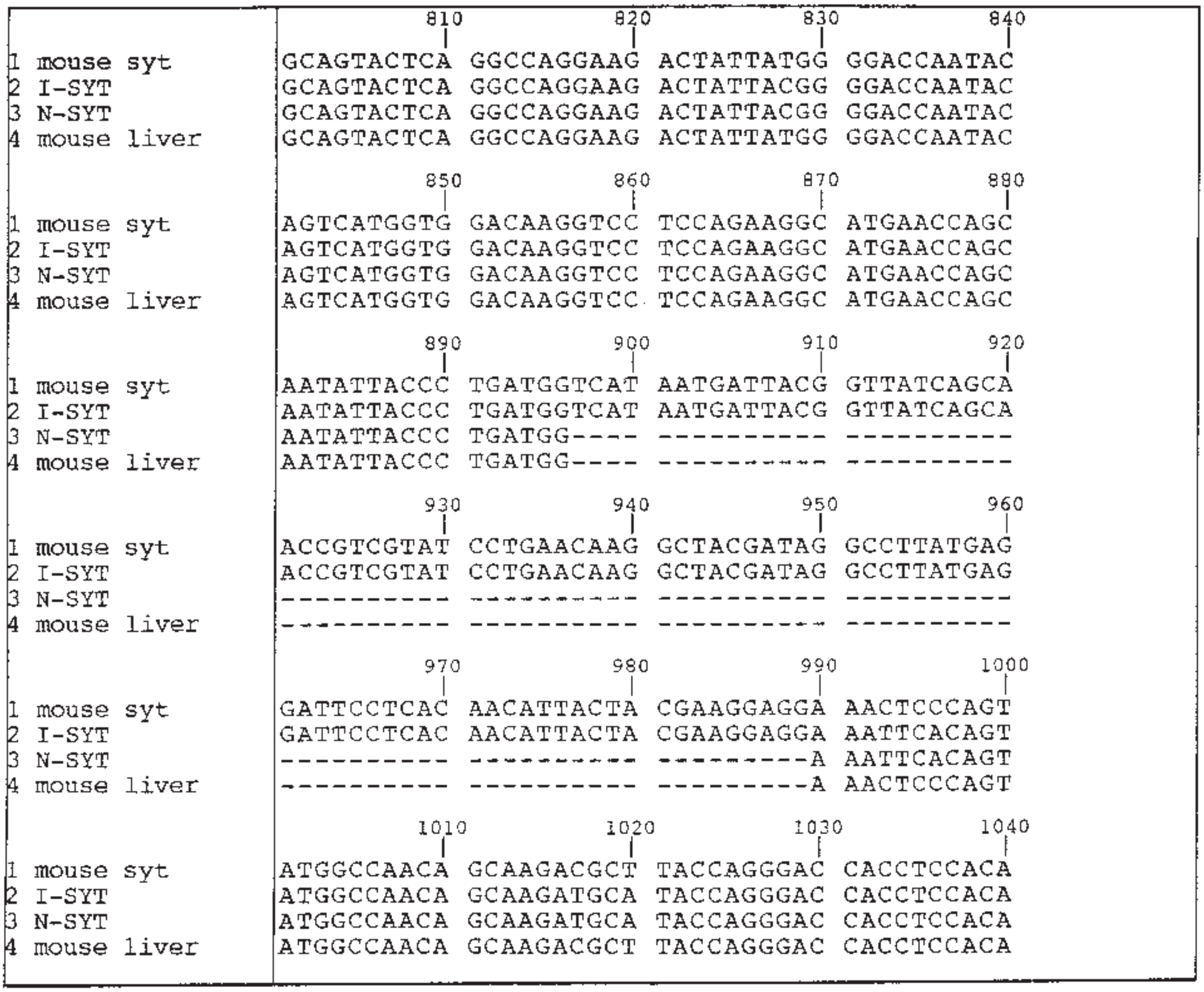

**Figure 1** (**C**) Alignment of SYT cDNAs sequences. Sequence 1: mouse SYT cDNA derived from NCBI data base (Accession no. X93357). Sequence 2: human I-SYT derived from normal synovia. Sequence 3: human N-SYT derived from normal synovia. Sequence 4: mouse N-SYT derived from a normal liver

### Analysis of a series of 59 synovial sarcomas

Following the detection of I-SYT and N-SYT forms in the normal tissues, we explored the SYT gene status in the series of 59 SSs already characterized for the presence of specific fusion transcripts. We performed PCR using SYT500F coupled to SSX1 for SYT-SSX1 fusion transcript, or SSX2 for SYT-SSX2, in order to selectively amplify the rearranged SYT gene only. For SYT-SSX4 and for tumour cases where no fusion transcript was detected, oligonucleotide SYT1000R was used. All 59 SSs and the CME cell line showed a coexpression of I-SYT and N-SYT (Figure 3), which we designated as I-SYT-SSX and N-SYT-SSX, respectively.

### Partial determination of exon/intron structure of SYT gene

A BLAST comparison of the novel 93 bp insertion sequence present in I-SYT form was made against HTGS data base of NCBI GenBank, retrieving clone RP11-737G21, Accession Number AC027229, which contains unordered genomic sequences of human chromosome 18.

This revealed that the 93 bp insertion was delimited by genomic sequences not detected in the SYT cDNA. In turn, these sequences were followed by the SYT cDNA portions present at the insertion site (between bp 885 and 886 of SYT cDNA) (Figure 4). This led us to postulate that the insertion represents another SYT exon, preceded and followed by introns of 2631 bp and 701 bp, respectively. This was confirmed by the finding of the canonic exon/intron donor acceptor consensus sequences present at the ends of introns.

### Densitometrical analysis

Analysis on normal tissue samples indicated a differential expression between N-SYT and I-SYT, the former showing always a greater intensity (Figure 2B). This was particularly true in the synovia, where 84.1% N-SYT vs 15.9% I-SYT was detected (Table 1).

In tumour cases a more heterogeneous expression pattern of I-SYT-SSX and N-SYT-SSX was observed, consistent with an increase of the I-SYT-SSX/N-SYT-SSX ratio (Table 2, column R), ranging from 0.34 to 13.7.

No significant correlation was observed between the SYT type and any type of these parameters: fusion transcript type, histological subtype, grade, tumour size, and source of the specimens (primary, relapse, metastasis) (data not shown).

 *© 2001 Cancer Research Campaign*

**Table 2** Summary of clinico-pathologic and molecular findings in 59 SSs.

| Case | Histologic subtype | Specimen type | Fusion transcript SYT-SSX | Densitrometical analysis | |
|---|---|---|---|---|---|
| | | | | %I-SYT-SSX / %N-SYT-SSX | Ratio (R) |
| 1 | Mo | M | 1 | | |
| 2 | Mo | R | 1 | 42.1 / 57.9 | 0.73 |
| 3 | Mo | M | 1 | 36 / 64 | 0.56 |
| 4 | Bi | M | 1 | 51.9 / 48.1 | 1.08 |
| 5 | Bi | R | 1 | | |
| 6 | Bi | P | 1 | 56.3 / 43.7 | 1.29 |
| 7 | Mo | P | 1 | | |
| 8 | Bi | P | 1 | 49 / 51 | 0.96 |
| 9 | Mo | R | 1 | | |
| 10 | Bi | R | 1 | 73 / 27 | 2.70 |
| 11 | Bi | P | 1 | 40.8 / 59.2 | 0.69 |
| 12 | Mo | P | 1 | 52.2 / 47.8 | 1.09 |
| 13 | Bi | M | 1 | 77 / 23 | 3.35 |
| 14 | Bi | P | 1 | | |
| 15 | Mo | P | 1 | 34.3 / 65.7 | 0.52 |
| 16 | Mo | R | 1 | 79.8 / 20.2 | 3.95 |
| 17 | Bi | P | 1 | 40.9 / 59.1 | 0.69 |
| 18 | Bi | M | 1 | 70 / 30 | 2.33 |
| 19 | Mo | M | 1 | | |
| 20 | Mo | M | 1 | | |
| 21 | Mo | R | 1 | 63.8 / 36.2 | 1.76 |
| 22 | Mo | M | 1 | | |
| 23 | Bi | P | 1 | 58.6 / 41.4 | 1.42 |
| 24 | Bi | R | 1 | 51 / 49 | 1.04 |
| 25a | Bi | R | 1 | 43.1 / 56.9 | 0.76 |
| 25b | Bi | M | 1 | 70.8 / 29.2 | 2.42 |
| 26 | Bi | M | 1 | 49.7 / 50.3 | 0.99 |
| 27a | Mo | P | 1 | 25.8 / 74.2 | 0.35 |
| 27b | Mo | M | 1 | 43.5 / 56.5 | 0.77 |
| 28a | Mo | M | 1 | 59.4 / 40.6 | 1.46 |
| 28b | Mo | M | 1 | 61 / 39 | 1.56 |
| 29 | Mo | M | 2 | 55.6 / 44.4 | 1.25 |
| 30 | Moo | P | 2 | 75.8 / 24.2 | 3.13 |
| 31 | Bi | R | 2 | 25.7 / 74.3 | 0.34 |
| 32 | Mo | R | 2 | 52.5 / 47.5 | 1.10 |
| 33 | Mo | P | 2 | 80.4 / 19.6 | 4.10 |
| 34 | Bi | P | 2 | 79.2 / 20.8 | 3.81 |

*© 2001 Cancer Research Campaign*

| | | | | | |
|---|---|---|---|---|---|
| 35 | Mo | R | 2 | 73<br>27 | 2.70 |
| 36 | Mo | P | 2 | 57<br>43 | 1.32 |
| 37 | Mo | R | 2 | | |
| 38 | Mo | P | 2 | | |
| 39 | Bi | P | 2 | | |
| 40 | Bi | M | 2 | | |
| 41a | Mo | M | 2 | 64.1<br>35.9 | 1.78 |
| 41b | Mo | M | 2 | 74.3<br>25.7 | 2.89 |
| 42a | Mo | P | 2 | 82<br>18 | 4.55 |
| 42b | Mo | M | 2 | 85.5<br>14.5 | 5.90 |
| 43 | Mo | M | 2 | 93.2<br>6.8 | 13.7 |
| 44 | Mo | P | 2 | 33.9<br>66.1 | 0.51 |
| 45 | Mo | M | 2 | 35.9<br>64.1 | 0.56 |
| 46 | Mo | P | 2 | 68.2<br>31.8 | 2.14 |
| 47 | Mo | M | 4 | | |
| 48 | Bi | M | | | |
| 49 | Mo | P | | | |
| 50 | Bi | P | | | |
| 47 | Mo | M | | | |
| 51 | Bi | M | | | |
| 52 | Mo | R | | | |
| 53 | Bi | P | | | |
| 54 | Bi | P | | | |
| 55a | Mo | R | | | |
| 55b | Mo | R | | | |
| 56 | Mo | M | | | |
| 57 | CME | - | 1 | 35<br>65 | 0.54 |

P = primary tumour, R = relapse, M = metastasis, Mo = monophasic subtype, Bi = biphasic subtype. Ratio is calculated as I-SYT/N-SYT percentage value.

### DISCUSSION

In this study 59 SSs (31 characterized by the non-random translocation SYT-SSX1, 16 by SYT-SSX2, one by SYT-SSX4, and 11 without a specific fusion transcript) were analysed. Morphologically, the series included 35 monophasic (16 with SYT-SSX1, 12 with SYT-SSX2, one with SYT-SSX4 and 6 without specific transcript) and 24 biphasic (15 with SYT-SSX1, 4 with SYT-SSX2 and 5 without specific transcript) tumours.

Following amplification of the whole cDNA from both SYT-SSX1 and SYT-SSX2 we observed that a novel SYT form, here designated I-SYT, was co-expressed with the usual normal SYT form, here named N-SYT, both of them fused to SSX genes. The novel SYT form showed an in-frame insertion of 93 bp which was 100% homologous with the mouse-corresponding sequence (de Bruijn et al, 1996). Co-expression of I-SYT and N-SYT was detected in all the analysed normal tissues (Figure 2B) according with the already published data regarding the mouse gene expression (de Bruijn et al, 1996).

Considering that this in-frame insertion was identical to the mouse sequence and that both the I-SYT and the N-SYT forms were present in normal tissues, we can conclude that SYT mRNA can undergo 2 alternative splicing events. This assumption is reinforced by BLAST comparison with genomic chromosome 18 sequences detected in NCBI HTGS GenBank database, which provided evidence that this 93 bp sequence is present in a genomic human clone. Moreover, the extremities of the I-SYT insertion sequence are clearly identifiable as exon donor and acceptor sites.

We found that the alternative splicing mechanism was maintained after the translocation of the X chromosome. In fact, both the I-SYT and the N-SYT forms were always detected after the fusion with one of the SSX gene family members, albeit in different amounts. More specifically, we observed that in normal tissues the expression levels were about 85% for N-SYT and 15% for I-SYT, suggesting a prevalent N-SYT expression, at least in this group (Table 1). Why the alternative splicing mechanism is driven toward N-SYT remains unknown. Most likely, the elevated percentage of N-SYT messenger was the cause leading to the isolation of only this human SYT sequence by Clark and co-workers (Clark et al, 1994).

By contrast, in tumour cases, the ratio (R) of I-SYT-SSX and N-SYT-SSX showed a variable increase in SSs, where the I-SYT-SSX expression level appeared enhanced over the all series. This value was calculated specifically on the fusion transcript

 © 2001 Cancer Research Campaign

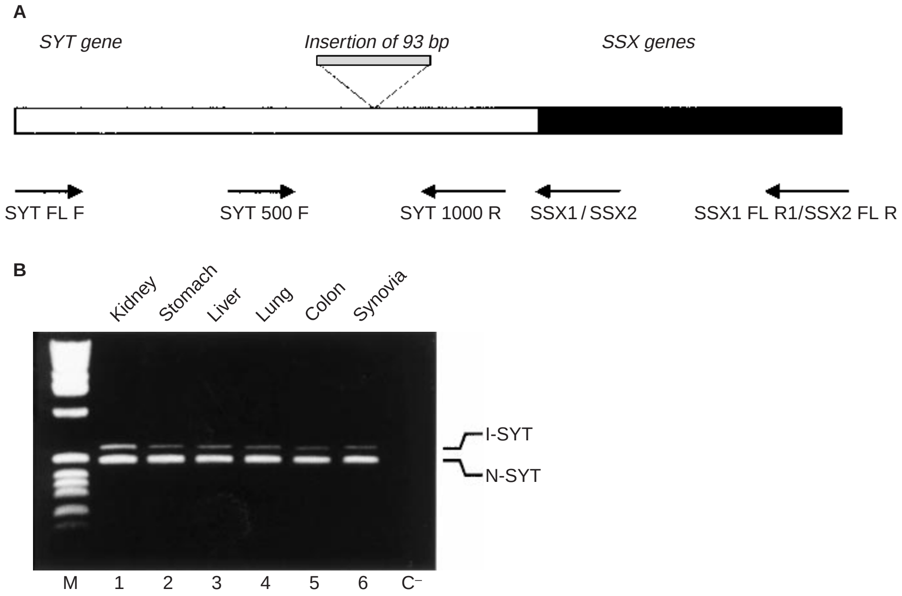

**Figure 2** (**A**) Schematic representation of the primers used in PCR reactions. (**B**) RT-PCR of SYT gene in normal tissues. The PCR reaction was performed using SYT500F and SYT 1000R. M = molecular weight marker. C⁻= no DNA was added to the PCR reaction

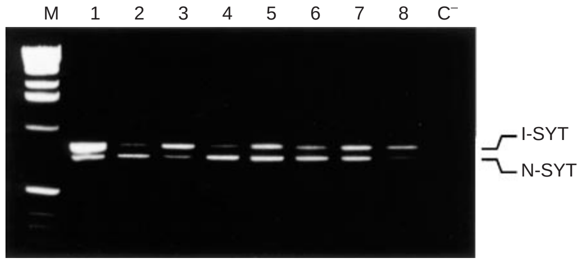

**Figure 3** RT-PCR of SYT-SSX in some tumour cases. The PCR reaction was performed using SYT500F and SSX1 for tumour cases with the SYT-SSX1 fusion transcript or SYT500F and SSX2 for tumour cases with the SYT-SSX2 fusion transcript. M = molecular weight marker. 1 = Case no. 16; 2 = Case no. 17; 3 = Case no. 18; 4 = Case no. 27a; 5 = Case no. 27b; 6 = Case n. 11; 7 = Case no. 12; 8 = Case no. 13 of Table 2. C⁻= no DNA was added to the PCR reaction messengers. In fact, in our PCR procedures we avoided the analysis of the non-rearranged allele that may derive from normal tissues and that may contaminate the tumour samples.

When we compared the primary tumour and the metastasis in the same patient, the R showed a constant increase in the metastatic tissue with respect to the primary one, at least in one informative case (Table 2, case n. 27) and this seemed to be maintained between the relapse and the metastasis (case n. 25 of Table 2) as well as between the subsequent metastases developed during tumour progression (case n. 41 of Table 2). This trend of expression is confirmed by real time PCR preliminary experiments that show an increased level of I-SYT in the analysed cases.

Biologically, the diversity in expression levels of the 2 SYT forms in normal and tumoral tissues could be due either to a different stability of the conformational structure of the chimaeric mRNA or to a prevalent oncogenic activity of I-SYT form. The I-SYT protein, in fact, contains 31 adjunctive residues (Figure 1B) with several microdomains rich in Q, Y, P and G, that are always present in a number of transcriptional co-activators (Tamkun et al, 1992; Strubin

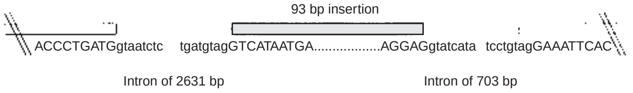

**Figure 4** Partial representation of Intron/Exon structure of human SYT gene. This structure was obtained comparing human I-SYT cDNA and a genomic DNA of chromosome 18 derived from NCBI data base (Accession no. AC027229). Exonic sequences are shown in uppercase letters, intronic in lowercase letters et al, 1995) and which may modulate the activity of the I-SYT protein. Functional studies are in progress to explore the in-vitro and in-vivo tumorigenic properties of the newly isolated I-SYT form.

### ACKNOWLEDGEMENTS

This work was supported by grants from ‘Ministero della Sanita’. Ricerca Finalizzata 1998-ICS030. 1/RF98.32-Italy’ and by AIRC (Associazione Italiana per la Ricerca sul Cancro), grant n.420.198.122. The authors are grateful to Dr Juan Rosai for the critical revision of the manuscript and thank Mr Mario Azzini for photographic assistance.

### REFERENCES

Antonescu CR, Kawai A, Leung DH, Lonardo F, Woodruff JM, Healey JH and Ladanyi M (2000) Strong association of SYT-SSX fusion type and morphologic epithelial differentiation in synovial sarcoma. *Diagn Mol Pathol* **9**: 1–8

Brett D, Whitehouse S, Per A, Shipley J, Cooper C and Goodwin G (1997) The SYT protein involved in the t(X;18) synovial sarcoma translocation is a transcriptional activator localized in nuclear bodies. *Hum Mol Gen* **6**: 1559–1564

Cielette MK, Socinski MA, Fletcher JA, Corson JM and Craighead JE (1994) Cardiac synovial sarcoma with translocation (X;18) associated with asbestos exposure. *Cancer* **73**: 74–78

Clark J, Roques PJ, Crew AJ, Gill S, Shipley J, Chan AM, Gustere BA and Cooper CS (1994) Identification of novel genes, SYT and SSX, involved in the t(X;18)(p11.2;q11.2) translocation found in human synovial sarcoma. *Nat Genet* **7**: 502–508

Crew AJ, Clark J, Fisher C, Grimed R, Chand A, Shipley J and Mitchell P (1995) Fusion in two genes, SSX1 and SSX2, encoding proteins with homology to the Krupper-associated box in human synovial sarcoma. *EMBO J* **14**: 2333–2340

de Bruijn D, Baats E, Zechner U, de Leeuw B, Balemans M, Olde Weghuis D, Hiring-Folz U and Geurts van Kessel A (1996) Isolation and characterization of the mouse homologue of SYT, a gene implicated in the development of human synovial sarcoma. *Oncogene* **13**: 643–648

DeLeeuw B, Balemans M and Geurts van Kessel A (1996) A novel Krupper-associated box containing SSX gene (SSX3) on the human X chromosome is not implicated in the t(X;18)-positive synovial sarcomas. *Cytogenet. Cell Genet* **73**: 179–183

Dos Santos NR, de Bruijn D, Balemans M, Janssen B, Gartner F, Manuel Lopez J, de Leeuw B and Geurts van Kessel A (1997) Nuclear localization of SYT, SSX, and the synovial sarcoma assiciated SYT-SSX fusion protein. *Human Mol Gen* **6**: 1549–1558

Enzinger FM and Weiss SW (eds) (1995) *Soft Tissue Tumors* 3d ed. St. Louis, MO: Mosby **29**: 757–786

Fetsh JF and Meis JM (1993) Synovial sarcoma of the abdominal wall. *Cancer* **72**: 469

Fisher C (1986) Synovial Sarcoma: Ultrastructural and immunohistochemical features of epithelial differentiation in monophasic and biphasic tumors. *Hum Path* **17**: 996–1008

Fritsch M, Epstein JI, Perlman EJ, Watts JC and Argani P (2000) *Hum Pathol* **31**: 246–250

Gure AO, Tureci O, Sahin U, Tsang S, Scanlan MJ, Jager E, Knuth A, Pfreundshuh M, Old LJ and Chen YT (1997) SSX: a multigene family with several members transcribed in normal testis and human cancer. *Int J Cancer* **72**: 965–997

Hisaoka M, Hashimoto H, Iwamasa T, Ishikawa K and Aoki T (1998) Primary synovial sarcoma of the lung: report of two cases confirmed by molecular detection of SYT-SSX fusion gene transcripts. *Histopathol* **34**: 205–210

Kashima T, Matsushita H and Kuroda M (1997) Biphasic synovial sarcoma of the peritoneal cavity with t(X;18) demonstrated by reverse transcriptase polymerase reaction. *Pathol Int* **47**: 637–641

Kawai A, Woodruff J, Healey JH, Brennan MF, Antonescu CR and Ladany M (1998) SYT-SSX gene fusion as a determinant of morphology and prognosis in synovial sarcoma. *N Engl J Med* **338**: 153–160

Lim FL, Soulez M, Koczan D, Thiesen HJ and Knight JC (1998) KRAB-related domain and a novel transcription repression domain in proteins encoded by SSX genes that are destrupted in human sarcomas. *Oncogene* **17**: 2013–2018

Mancuso T, Mezzelani A, Riva C, Fabbri A, Dal Bo L, Sampietro G, Perego P, Casali P, Zunino F, Sozzi G, Pierotti MA and Pilotti S (2000) Analysis of SYT-SSX fusion transcript and bcl2 expression and phosphorylation status in synovial sarcoma. *Lab Invest* **80**: 805–813

Miloro M, Quinn PD and Stewart J (1994) Monophasic spindle sell synovial sarcoma of the head and neck: report of two cases and review of the litterature. *J Oral Maxillofac Surg* **52**: 309–313

Nicholson AG, Goldstraw P and Fisher C (1998) Synovial sarcoma of the pleura and its differentiation from other primary pleural tumours: a clinicopathological and immunohistochemical review of three cases. *Histopathol* **33**: 508–513

Nielsen GP, Shaw PA, Rosenberg AE, Dickersin GR, Young RH and Scully RE (1996) Synovial sarcoma of the vulva: a report of two cases. *Mod Pathol* **9**: 970–974

Nilsson G, Skytting B, Xie Y, Brodin B, Perfekt R, Mandahl N, Lundeberg J, Uhlen M and Larsson O (1999) The SYT-SSX1 variant of synovial sarcoma is associated with a high rate of tumor cell proliferation and poor clinical outcame. *Can Res* **59**: 180–184

Renwick PJ, Reeves BR, Dal Cin P, Fletcher CD, Kempski H, Sciot R, Kazmirerczak B, Jani K, Sonobe H and Knight JC (1995) Two categories of synovial sarcoma defined by divergent chromosome translocation breckpoints in Xp11.2, with implications for histologic subclassification of synovial sarcoma. *Cytogenet Cell Genet* **70**: 58–63

Skytting B, Nilsson B, Brodin B, Xie Y, Lundeberg J, Uhlen M and Larsson O (1999) A novel fusion gene, SYT-SSX4, in synovial sarcoma. *J Natl Cancer Inst* **91**: 974–975

Strubin M, Newel JM and Matthias P (1995) OBF-1, a novel B cell-specific coactivator that stimulated immunoglobulin promoter activity through association with octamer-binding proteins. *Cell* **80**: 497–506

Turc-Carel C, Dal Cin P, Limon J, Rao U, Li FP, Corson JM, Zimmermann R, Parry DM, Cowan JM and Sandberg AA (1987) Involvement of chromosome 18 in primary cytogenetic change in human neoplasia: nonrandom translocation in synovial sarcoma. *Proc Nat Acad Sci USA* **84**: 1981–1985

Tamkun JW, Deuring R, Scott MP, Kissinger M, Pattatucci AM, Kaufman TC and Kennison JA (1992) Brahama: a regulatory of Drosophila homeotic genes structurally related to the yeast transcriptional activator SNF/SW12. *Cell* **68**: 561–571

Tsuji S, Hisakoa M and Morimitsu Y (1998) Detection of SYT-SSX fusion transcripts in synovial sarcoma by reverse transcription-polymerase chain reaction using archival paraffin embedded tissues. *Am J Pathol* **153**: 1807–1812

Zeren H, Moran CA, Suster S, Fishback NF and Koss MN (1995) Primary pulmonary sarcomas with features of monophasic synovial sarcoma. A clinicopathological, immunohistochemical and ultrastructural study of 25 cases. *Hum Pathol* **26**: 474–480

 *© 2001 Cancer Research Campaign*
